# Fully solution-processed phase-pure 3D/2D perovskite bilayer heterojunctions

**DOI:** 10.1038/s42004-023-00812-w

**Published:** 2023-01-13

**Authors:** Xinxin Lian, Hong Zhang, Xiaoliang Mo, Junhao Chu

**Affiliations:** Department of Materials Science, Institute of Optoelectronics, Shanghai Frontiers Science Research Base of Intelligent Optoelectronics and Perception, Fudan University, 200433 Shanghai, China

**Keywords:** Photovoltaics, Solar cells, Materials chemistry

## Abstract

Combining the superior photovoltaic performance of three-dimensional perovskites and the intrinsic durability of two-dimensional perovskites, the construction of 3D/2D perovskite bilayer heterojunctions is a promising strategy to realize efficient and stable perovskite solar cells, but it is still a challenge to control the phase purity, film thickness, orientation, and crystal structure of 2D perovskites. Now, a solution-processing strategy has overcome this challenge by directly coating a tailored single-crystal 2D perovskite ink on as-prepared 3D perovskite films, resulting in effective, ultra-stable and phase-pure 3D/2D perovskite bilayer heterojunctions.

Driving the world with sunlight is beneficial to achieving carbon peak in 2030 and carbon neutrality in 2060^1^. It is of vital significance to develop efficient and stable photoelectric light-harvesting materials. Organic–inorganic metal halide perovskites have become a star material in the photovoltaic field due to their high optical absorption coefficient, tunable band gap, long carrier lifetimes, and low exciton binding energy. Within a decade, the highest certified efficiency of perovskite solar cells (PSCs) has rapidly increased to 25.7%^2^, which is comparable to that of monocrystalline silicon solar cells. However, the interface between a perovskite and a charge transfer layer contains a high concentration of defects, which greatly reduces the long-term stability of PSCs^3^. Research endeavors are focused on the development of composition engineering^4^, additive engineering^5^, solvent engineering^6^, interfacial passivation^7^ and processing methods^8^. In particular, 3D/2D perovskite bilayer heterojunctions have been widely shown to achieve efficient and stable PSCs by controlling interfacial defect density and energy band alignment at the interface of perovskite/charge transport layers^9^. However, it is challenging to control phase purity, film thickness, orientation, and crystal structure of 2D perovskites in traditional fabrication processes, i.e., by coating a 2D spacer (alkylammonium cation) solution on as-prepared 3D perovskite films (Fig. 1A). Meanwhile, the solvent of the 2D spacer (e.g., isopropanol) easily degrades the underlying 3D perovskite film.

**Fig. 1:**
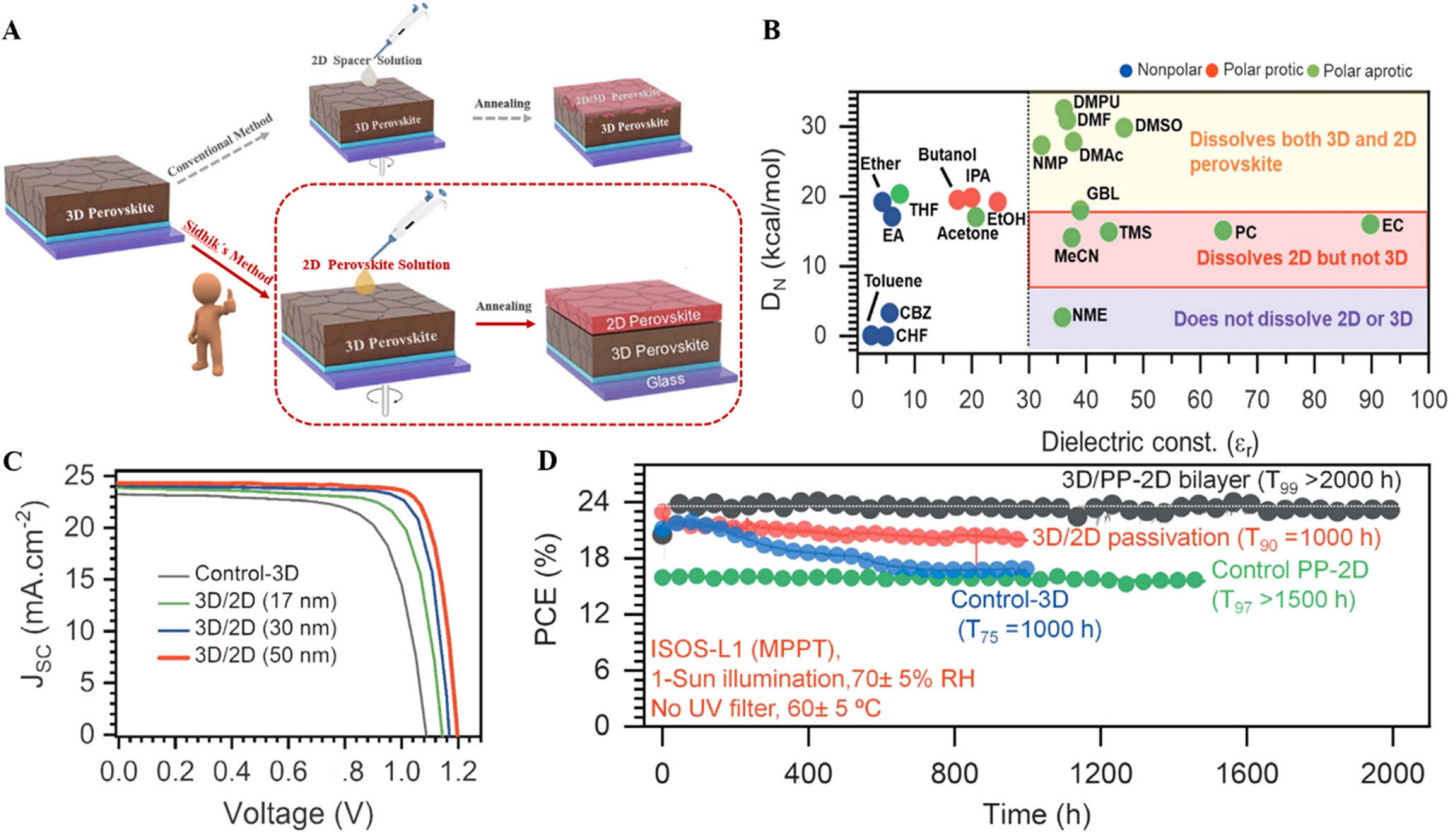
Preparation of 2D/3D perovskite bilayer heterojunctions and performance comparison of different methods and solvents. **A** Schematic illustration of preparing 3D/2D perovskite bilayer heterojunctions with different methods. **B** Comparison of different solvents based on dielectric constant (Ɛ_r_) and Gutmann number (D_N_). **C** Current–voltage curves of the champion 3D/2D perovskite bilayer heterojunction PSCs. **D** Stability assessment of the 3D/2D-based modules with different perovskite films at maximum power point tracking in an ambient atmosphere. **A** Adapted and (**B**–**D**) reproduced from *Science* **377**, 1425–1430 (2022). Reprinted with permission from AAAS.

Writing in *Science*, a team led by Aditya Mohite from Rice University, USA, and Jacky Even from the University of Rennes, France, report a strategy to fabricate effective, ultrastable and phase-pure 3D/2D perovskite bilayer heterojunctions by directly coating single-crystal 2D perovskite inks on as-prepared 3D perovskite films (https://www.science.org/doi/10.1126/science.abq7652)^10^. This strategy was realized by tailoring an orthogonal solvent system with a suitable dielectric constant (Ɛ_r_) and Gutmann donor number (D_N_) for dissolving 2D perovskite single crystals, while avoiding dissolution of the underlying 3D perovskite layer. “The biggest challenge was understanding the solvent interactions and solvation dynamics in order to find the right solvent”, comments Mohite. They found that the 2D perovskite single-crystal powders can be effectively dissolved without dissolving or degrading the underlying 3D perovskite film when Ɛ_r_ > 30 and 5 < D_N_ < 18 kcal mol^−1^ (Fig. 1B). Considering the solubility and low-temperature preparation of 2D (BA_2_MA_2_Pb_3_I_10_) and 3D (Cs_0.05_MA_0.10_FA_0.85_Pb(I_0.90_Br_0.10_)_3_) perovskites, highly volatile acetonitrile (MeCN; boiling point ≈82 °C) was selected for fabricating the target 3D/2D perovskite bilayer heterojunctions. Different phase-pure 2D BA_2_MA_n-1_Pb_n_I_3n+1_ perovskites (*n* = 1–4) were evenly distributed on the surface of the 3D perovskite with a sharp interface transition between the 2D and 3D perovskites, which indicates that the solvent has negligible influence on the underlying 3D perovskite. The 3D/2D layers tune the energy-level alignment. Additionally, interfacial non-radiative recombination at the 3D perovskite/hole transport layer interface is suppressed after the introduction of 2D BA_2_MA_2_Pb_3_I_10_, which is an ideal hole extraction and electron barrier layer. The 2D perovskites also enable grain boundary passivation and reduce residual PbI_2_ on the surface of the 3D perovskite. “We have shown that 2D perovskites can not only be used for passivation, but can have a synergistic effect on the photogeneration capability, and the stability,” says Mohite. “We observed a surprisingly high stability of 2000 h running photovoltaic performance with an efficiency of ∼24%, which is a big step towards commercialization of these perovskite devices.”

“Scaling up of the 3D/phase-pure 2D bilayer structures should be the immediate step which will help to realize commercially viable perovskite solar cell modules,” explains Mohite. Their work indeed provides such a scalable solution-processing method to fabricate phase-pure, thick (>30 nm) and fully covered 2D perovskite interfacial layers. The strategy is also feasible in p-i-n devices, demonstrating its broad applicability. However, the growth mechanisms of phase-pure 2D perovskite upper layers and the crystal structures of the 3D/2D perovskite bilayer heterojunctions are still not entirely clear. Meanwhile, more in-depth research by a combination of interface engineering, composition engineering and solvent engineering should be conducted to achieve more efficient and operationally stable perovskite photovoltaics.
